# HPV overtakes smoking as the leading cause of oropharyngeal cancer in Ireland: experience of a head and neck surgery tertiary referral centre

**DOI:** 10.1007/s11845-024-03715-4

**Published:** 2024-05-28

**Authors:** Eoin F. Cleere, Josh Murphy, Thomas J. Crotty, Justin M. Hintze, Conrad V. I. Timon, John Kinsella, Conall W. R. Fitzgerald, Paul Lennon

**Affiliations:** Department of Otolaryngology Head and Neck Surgery, St James’s Hospital, Dublin, Ireland

**Keywords:** Epidemiology, HPV, Human papillomavirus, Oropharyngeal cancer, Squamous cell carcinoma

## Abstract

**Background:**

Worldwide, the incidence of oropharyngeal squamous cell carcinoma (OPSCC) caused by human papillomavirus (HPV), a sexually transmitted virus, is increasing. This increase has yet to be demonstrated in an Irish cohort.

**Aims:**

To evaluate the number of OPSCC presentations locally, to stratify cases by HPV status and to estimate if any changes in the patient population had occurred over a 10-year period.

**Methods:**

A STROBE-compliant, retrospective evaluation of patients with OPSCC at St James’s Hospital between 2012 and 2022 was performed. Patients with non-SCC histology, undocumented HPV status and residual or recurrent tumours were excluded.

**Results:**

We included 294 patients with a mean age of 60.4 years (95% CI 59.2–61.5 years) and 175 (59.5%) patients had HPV+ OPSCC. The number of new OPSCC diagnoses increased from 115 patients (39.1%) between 2012 and 2016 to 179 patients (60.9%) between 2017 and 2021. This was associated with an increased proportion of HPV-linked OPSCC (50.4% 2012–2016 vs. 65.4% 2017–2021, *p* = 0.011). Over time, more patients had a functionally limiting comorbidity (*p* = 0.011). The mean age of HPV+ OPSCC cases increased by 3.6 years (*p* = 0.019). Patients with HPV+ OPSCC had greater 2-year OS (83.9% vs. 54.9%; *p* < 0.001) and 2-year DFS (73.5% vs. 45.6%; *p* < 0.001). The 2-year OS and DFS did not change over time for HPV+ or HPV− patients.

**Conclusions:**

In our institution, the number of patients with OPSCC is increasing due to an escalation in cases associated with HPV. Population-level interventions such as vaccination programs may alter the current increase in the incidence of these tumours.

## Introduction

Globally, almost 100,000 patients were diagnosed with oropharyngeal squamous cell carcinoma (OPSCC) in 2020 while it was responsible for 48,100 deaths [1]. Over the last 20–30 years, the incidence of OPSCC has increased markedly while the incidence of other head and neck cancers has either remained steady or fallen [2]. Historically, like most head and neck cancers, OPSCC was largely due to the synergistic effects of tobacco and alcohol usage. However, around the turn of the century, it became clear that the increase in OPSCC was not derived from an increase in patients who smoke and drink heavily. Landmark work by Gillison et al. demonstrated human papillomavirus (HPV), a sexually transmitted virus, as an oncogenic driver in a subset of molecularly distinct head and neck cancer patients, particularly in OPSCC [3]. Subsequent analysis regarding epidemiological trends in OPSCC revealed an increased incidence of HPV-related OPSCCs was responsible for the increasing incidence of OPSCC overall [4–6].

Following on from this, it became evident that patients with HPV-mediated OPSCC (HPV+ OPSCC) were younger and less likely to smoke or drink alcohol versus non-HPV-mediated tumours (HPV− OPSCC) [7]. In addition, these patients experienced markedly improved outcomes compared with their HPV− OPSCC counterparts [8, 9]. As such, these molecularly and epidemiologically distinct tumours are now considered separate entities with separate staging systems [10].

National data in Ireland has detailed that the number of OPSCCs has also increased from 59 cases in 1994 to 143 cases in 2014, similar to international trends [11]. However, to date, no study has evaluated if this increase has been mediated by an increasing proportion of HPV+ OPSCC. Thus, the primary aim of the present study was to evaluate patients over a 10-year period presenting with OPSCC to our institution and to assess if the number of cases of OPSCC was increasing. Secondarily, we wanted to assess if any changes in local presentations were mediated by HPV+ OPSCC and if our patient demographics had changed in accordance with this. Finally, we looked to evaluate overall survival (OS) and disease-free survival (DFS) in patients presenting with OPSCC to our institution.

## Methods

### Study design and patient selection

A single centre, retrospective observational study was performed in accordance with the STrengthening the Reporting of OBservational studies in Epidemiology (STROBE) checklist [12]. Local institutional ethical approval was obtained at St James’s Hospital prior to the study. All patients with a histologically diagnosed OPSCC discussed at the St James’s Hospital head and neck multidisciplinary team meeting between January 2012 and December 2021 were considered for inclusion. Cases were identified from a prospectively maintained institutional oropharyngeal cancer database. January 2012 was selected to begin the study period as prior to this not all new OPSCC diagnoses underwent routine HPV testing at our institution.

To be eligible, patients were required to be older than 18 years old at the time of multidisciplinary discussion. Additionally, patients were required to have a documented HPV status on their diagnostic biopsy specimen. Patients were excluded for the following reasons: (1) non-SCC histology, (2) no documented P16 or HPV testing of tumour specimens, (3) no treatment or follow-up data available and (4) residual or recurrent oropharyngeal tumours.

### Data collection and variables

Data was collected retrospectively from patient healthcare records by three authors (E.F.C., J.M. and T.J.C.). Patient demographics, tumour location, staging and HPV status were as recorded at time of presentation. Frailty was recorded as a dichotomous outcome using the five-item modified Frailty Index [13] (5mFI—scores of 2 or greater equating to frail) and the severity of patient comorbidities was classified using the American Society of Anaesthesiologists (ASA) classification [14]. The ASA classification was recorded as a dichotomous outcome with a score of 3 or more as a cut-off (ASA of 3 = comorbidity leading to functional limitation). Patients were considered a smoker if they had a greater than 10 pack-year history recorded while alcohol usage was recorded as a dichotomous outcome. For the purposes of this study, tumour staging was performed using the American Joint Committee on Cancer (AJCC) staging 7th edition. The more recent 8th edition of AJCC staging now incorporates HPV-positive OPSCC as a separate entity with its own staging system. As such, we used the AJCC 7th edition to allow direct comparisons between HPV-positive and HPV-negative tumours. Our local institutional protocol uses P16 testing as a surrogate marker of HPV status in OPSCC which is an established practice worldwide [15, 16]. Treatment received and follow-up outcomes were recorded directly from patient records.

### Statistical analysis

Statistical analysis was carried out using STATA BE version 17.0. Basic descriptive statistics were performed with continuous variables displayed as mean values with associated 95% confidence intervals (CIs). Statistical tests performed included Kruskal–Wallis or chi-squared test as appropriate. For the purposes of temporal analysis (i.e. changes over time), patients were grouped into two groups: group 1 diagnosed between 2012 and 2016 and group 2 between 2017 and 2021. Survival outcomes included overall survival (OS) (death from any cause) and disease-free survival (DFS) (either tumour recurrence or death from any cause). The Kaplan–Meier method was used for analysis of survival outcomes with differences between groups estimated using the log-rank test. For the purpose of statistical analysis, a *p* value of less than 0.05 was considered significant.

## Results

### Patient characteristics

Two-hundred and ninety-four patients were included in the final analysis with a mean age of 60.4 years (95% CI 59.2–61.5 years). Over three-quarters of patients were male (234/294, 79.6%). The majority of patients had a history of smoking (194/294, 66.0%) and regularly consumed alcohol (218/294, 74.2%). One-hundred and seventy-five (59.5%) patients had HPV+ OPSCC while 119 patients (40.5%) had HPV− OPSCC (Table 1). The majority of patients had advanced disease at presentation with 85.4% (251/294) having stage III/IV disease. Combination chemoradiotherapy was the most frequent curative intent treatment modality (227/294, 77.2%) followed by single modality radiotherapy (26/294, 8.8%) with 21 patients receiving palliative intent treatment (7.1%) (Table 1).

**Table 1:** OPSCC patient characteristics, 2012–2021

**Characteristic**	**Total cohort (*n*** **= 294)**	**HPV− (*n*** **= 119)**	**HPV+ (*n*** **= 175)**	***p*** value*
Mean age (95% CI)	60.4 years (59.2–61.5 years)	62.2 years (60.4–64.1 years)	59.1 years (57.6–60.5 years)	**0.004**^**a**^
Male	234 (79.6%)	85 (71.4%)	149 (85.1%)	**0.004**
Female	60 (20.4%)	34 (28.6%)	26 (14.9%)	
Smoking	194 (66.0%)	105 (88.2%)	89 (50.9%)	** < 0.001**
Alcohol	218 (74.2%)	100 (84.0%)	118 (67.4%)	**0.005**
5mFI				**0.002**
*0–1*	220 (74.8%)	78 (65.6%)	142 (81.1%)	
*2–5*	74 (25.2%)	47 (34.4%)	33 (18.9%)	
ASA				** < 0.001**
1–2	207 (70.4%)	63 (55.5%)	141 (80.6%)	
3 +	87 (29.6%)	53 (44.5%)	34 (19.4%)	
Primary site				** < 0.001**
*Tonsil*	143 (48.6%)	45 (37.8%)	98 (56.0%)	
*BOT*	102 (34.7%)	36 (30.3%)	66 (34.7%)	
*Other*	49 (16.7%)	38 (31.9%)	11 (6.3%)	
AJCC7 stage				0.980
*I/II*	43 (14.6%)	18 (15.1%)	25 (14.3%)	
*III/IVa/IVb*	241 (82.0%)	97 (81.5%)	144 (82.3%)	
*IVc*	10 (3.4%)	4 (3.4%)	6 (3.4%)	
T stage				**0.002**
*1*	42 (14.3%)	16 (13.5%)	26 (14.9%)	
*2*	99 (33.7%)	26 (21.9%)	73 (41.7%)	
*3*	82 (27.9%)	37 (31.1%)	45 (25.7%)	
*4*	71 (24.1%)	40 (33.5%)	31 (17.7%)	
N stage				** < 0.001**
*0*	67 (22.8%)	39 (32.8%)	28 (16.0%)	
+	227 (77.2%)	80 (67.2%)	147 (84.0%)	
Treatment				** < 0.001**
*RT*	26 (8.8%)	13 (10.9%)	13 (7.4%)	
*CRT*	227 (77.2%)	74 (62.2%)	153 (87.4%)	
*Surgery only*	16 (5.4%)	12 (10.1%)	4 (2.3%)	
*Surgery* + *adjuvant*	4 (1.3%)	4 (3.4%)	0 (0.0%)	
*Palliation*	21 (7.1%)	16 (13.5%)	5 (2.9%)	

*95% CI* 95% confidence interval, *5mFI* 5-item Modified Frailty Index, *ASA* American Society of Anaesthesiologists classification, *BOT* base of tongue, *Other* soft palate or posterior oropharyngeal wall, *AJCC* American Joint Committee on Cancer, *RT* radiotherapy, *CRT* chemoradiotherapy

*Chi-square test unless specified

^a^Kruskal-Wallis test

Patients with HPV+ OPSCC were significantly younger (59.1 years vs. 62.2 years; *p* = 0.004) and less likely to have a smoking history (50.9% vs. 88.2%; *p* < 0.001) or regularly consume alcohol (67.4% vs. 84.0%; *p* = 0.005). Additionally, HPV+ OPSCC patients were less likely to be classified as frail using the 5mFI (HPV+ 33/175, 19.4% vs. HPV− 47/115, 34.4%; *p* = 0.002) or have an ASA score of 3 or more (HPV+ 34/175, 19.4% vs. HPV− 53/119, 44.5%; *p* < 0.001). Patients with HPV+ OPSCC were more likely to have primary tumours affecting the palatine tonsils or base of the tongue (*p* < 0.001). Overall, AJCC 7th edition staging did not differ by HPV status (*p* = 0.980) although patients with HPV− OPSCC had more advanced T classifications (*p* = 0.002) while patients with HPV+ OPSCC were more likely to have nodal disease at presentation (HPV+ 147/175, 84.0% vs. HPV− 80/119, 67.2%; *p* < 0.001) (Table 1).

### Changes in patient cohort over time

**Fig. 1:**
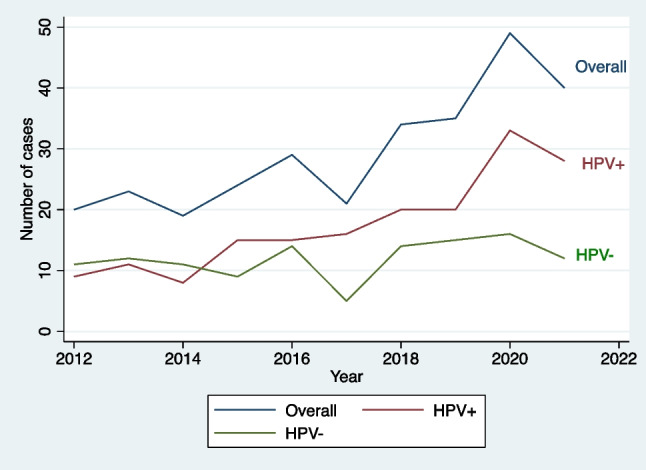
Number of cases presenting with OPSCC each year 2012–2021

The number of presentations with OPSCC at our institution increased over the study period (Fig. 1). Between 2012 and 2016, 115 patients (39.1%) were diagnosed and this increased to 179 patients (60.9%) in 2017–2021. This was associated with a rise in the number of HPV+ OPSCC cases but no change in the number of HPV− OPSCC cases with the proportion of OPSCC due to HPV increasing from 50.4% in 2012–2016 to 65.4% in 2017–2021 (*p* = 0.011) (Fig. 1). The mean age of patients with OPSCC increased over time from 59.1 years (95% CI 57.1–61.1 years) in 2012–2016 to 61.2 years (95% CI 59.8–62.6 years) between 2017 and 2021 (*p* = 0.043). Additionally, the proportion of patients with an ASA score of 3 or more increased over time (2012–2016 23.5% vs. 2017–2021 33.5%; *p* = 0.011) (Table 2).

**Table 2:** Patient characteristics by year of diagnosis

**Characteristic**	**2012–2016 (*n*** **= 115)**	**2017–2021 (*n*** **= 179)**	***p*** value*
Mean age (95% CI)	59.1 years (57.1–61.1 years)	61.2 years (59.8–62.6 years)	**0.043**^**a**^
Male	88 (76.5%)	146 (81.6%)	0.295
Female	27 (23.5%)	33 (18.4%)	
Smoking	78 (67.8%)	116 (64.8%)	0.379
Alcohol	87 (75.6%)	131 (73.8%)	0.243
5mFI			0.592
*0–1*	88 (76.5%)	132 (73.7%)	
*2–5*	27 (23.5%)	47 (26.3%)	
ASA			**0.011**
1–2	88 (76.5%)	119 (66.5%)	
3+	27 (23.5%)	60 (33.5%)	
HPV status			**0.011**
*Positive*	58 (50.4%)	117 (65.4%)	
*Negative*	57 (49.5%)	62 (34.6%)	
Primary site			0.114
*Tonsil*	56 (48.7%)	87 (48.6%)	
*BOT*	34 (29.6%)	68 (38.0%)	
*Other*	25 (21.7%)	24 (13.4%)	
AJCC7 stage			0.264
*I/II*	12 (10.4%)	31 (17.3%)	
*III/IVa/IVb*	99 (86.1%)	142 (79.3%)	
*IVc*	4 (3.5%)	6 (3.4%)	
T stage			0.937
*1*	17 (14.8%)	25 (14.0%)	
*2*	40 (34.8%)	59 (33.0%)	
*3*	31 (26.9%)	51 (28.5%)	
*4*	27 (23.5%)	44 (24.5%)	
N stage			0.529
*0*	24 (20.9%)	43 (24.0%)	
+	91 (79.1%)	136 (76.0%)	
Treatment			0.115
*RT*	5 (4.4%)	21 (11.7%)	
*CRT*	90 (78.3%)	137 (76.5%)	
*Surgery only*	6 (5.2%)	10 (5.6%)	
*Surgery* + *adjuvant*	2 (1.7%)	2 (1.1%)	
*Palliation*	12 (10.4%)	9 (5.0%)	

*95% CI* 95% confidence interval, *5mFI* 5-item Modified Frailty Index, *ASA* American Society of Anaesthesiologists classification, *BOT* base of tongue, *Other* soft palate or posterior oropharyngeal wall, *AJCC* American Joint Committee on Cancer, *RT* radiotherapy, *CRT* chemoradiotherapy

*Chi-square test unless specified

^a^Kruskal-Wallis test

Patients were subsequently grouped into HPV+ and HPV− OPSCC groups. Over time, the mean age of patients with HPV+ OPSCC increased from 56.6 years (95% CI 54.0–59.3 years) between 2012 and 2016 to 60.2 years (95% CI 58.5–62.0 years) between 2017 and 2021 (*p* = 0.019). The distribution of patient staging altered over time in the HPV+ OPSCC group with more patients having either stage I/II or metastatic disease at time of presentation in the 2017–2021 period (*p* = 0.023). A greater proportion of HPV− OPSCC patients had an ASA score of 3 or more over time (2012–2016 35.1% vs. 2017–2021 53.2%; *p* = 0.046). Other characteristics did not change over time in the either the HPV+ or HPV− OPSCC cohorts (Table 3).

**Table 3:** Patient characteristics over time stratified by HPV status

	**HPV− OPSCC**			**HPV+ OPSCC**		
**Characteristic**	**2012–2016 (*n* = 57)**	**2017–2021 (*n* = 62)**	*p* value*	**2012–2016 (*n* = 58)**	**2017–2021 (*n* = 117)**	*p* value*
Mean age (95% CI)	61.6 years (58.6–64.6 years)	62.8 years (60.5–65.2 years)	0.738^a^	56.6 years (54.0–59.3 years)	60.2 years (58.5–62.0 years)	**0.019**^**a**^
Male	41 (71.9%)	44 (70.9%)	0.908	47 (81.1%)	102 (87.2%)	0.282
Female	16 (28.1%)	18 (29.1%)		11 (18.9%)	15 (12.8%)	
Smoking	47 (82.5%)	58 (93.5%)	0.065	31 (53.5%)	58 (49.6%)	0.125
Alcohol	46 (80.7%)	54 (87.1%)	0.535	41 (70.7%)	77 (65.8%)	0.176
5mFI			0.308			0.700
*0–1*	40 (70.2%)	38 (61.3%)		48 (82.8%)	94 (80.3%)	
*2–5*	17 (29.8%)	24 (38.7%)		10 (17.2%)	23 (19.7%)	
ASA			**0.046**			0.083
1–2	37 (64.9%)	29 (46.8%)		51 (87.9%)	90 (76.8%)	
3+	20 (35.1%)	33 (53.2%)		7 (12.1%)	27 (23.2%)	
Primary site			0.986			**0.042**
*Tonsil*	22 (38.6%)	23 (37.1%)		34 (58.6%)	64 (54.7%)	
*BOT*	17 (29.8%)	19 (30.6%)		17 (29.3%)	49 (41.9%)	
*Other*	18 (31.6%)	20 (32.3%)		7 (12.1%)	4 (3.4%)	
AJCC7 stage			0.104			**0.023**
*I/II*	8 (14.0%)	10 (16.1%)		4 (6.9%)	21 (17.9%)	
*III/IVa/IVb*	45 (79.0%)	52 (83.9%)		54 (93.1%)	90 (76.9%)	
*IVc*	4 (7.0%)	0 (0.0%)		0 (0.0%)	6 (5.1%)	
T stage			0.841			0.908
*1*	8 (14.0%)	8 (12.9%)		9 (15.5%)	17 (14.5%)	
*2*	14 (24.6%)	12 (19.4%)		26 (44.8%)	47 (40.2%)	
*3*	17 (29.8%)	20 (32.3%)		14 (24.1%)	31 (26.5%)	
*4*	18 (31.6%)	22 (35.3%)		9 (15.5%)	22 (18.8%)	
N stage			0.790			0.151
*0*	18 (31.6%)	21 (33.9%)		6 (10.3%)	22 (18.8%)	
+	39 (68.4%)	41 (64.1%)		52 (89.7%)	95 (81.2%)	
Treatment			0.252			0.170
*RT*	4 (7.0%)	9 (14.5%)		1 (1.7%)	12 (10.3%)	
*CRT*	36 (63.2%)	38 (61.3%)		54 (93.1%)	99 (84.6%)	
*Surgery only*	4 (7.0%)	8 (12.9%)		2 (3.5%)	2 (1.7%)	
*Surgery* + *adjuvant*	2 (3.5%)	2 (3.2%)		0 (0.0%)	0 (0.0%)	
*Palliation*	11 (19.3%)	8 (8.1%)		1 (1.7%)	4 (3.4%)	

*95% CI* 95% confidence interval, *5mFI* 5-item Modified Frailty Index, *ASA* American Society of Anaesthesiologists classification, *BOT* base of tongue, *Other* soft palate or posterior oropharyngeal wall, *AJCC* American Joint Committee on Cancer, *RT* radiotherapy, *CRT* chemoradiotherapy

*Chi-square test unless specified

^a^Kruskal-Wallis test

### Survival outcomes

**Fig. 2:**
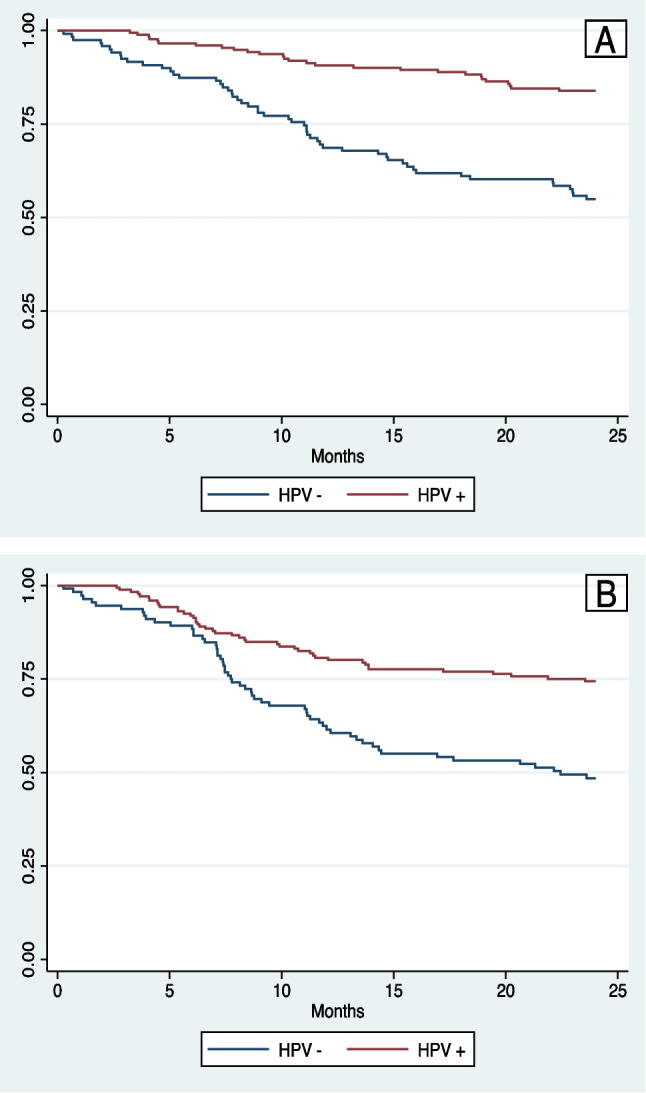
**A** Two-year overall survival stratified by HPV status. **B** Two-year disease-free survival stratified by HPV status

**Fig. 3:**
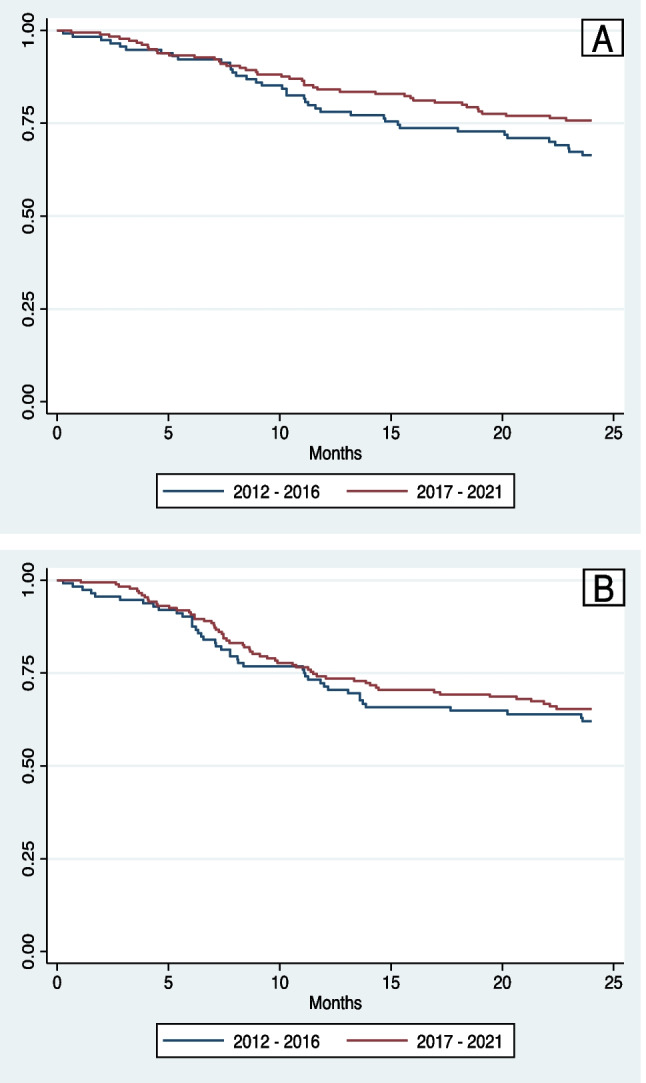
**A** Two-year overall survival stratified by year of diagnosis. **B** Two-year disease-free survival stratified by year of diagnosis

**Fig. 4:**
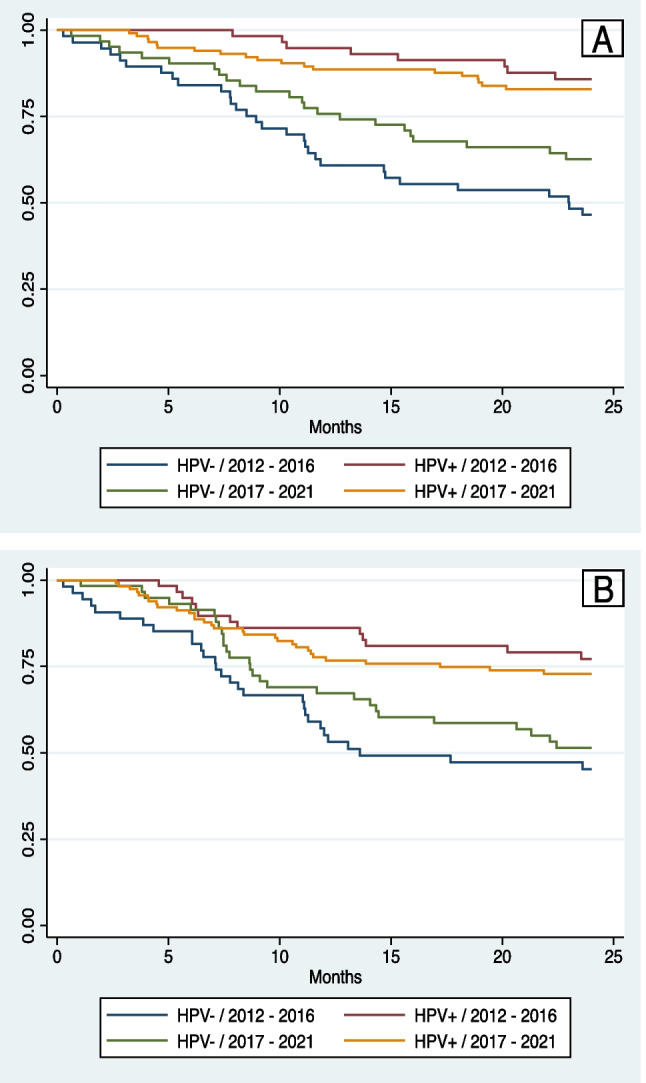
**A** Two-year overall survival stratified by year of diagnosis and HPV status. **B** Two-year disease-free survival stratified by year of diagnosis and HPV status

At 2 years, the OS for all patients was 72.0% (95% CI 66.4–76.8%) and the 2-year DFS was 62.1% (95% CI 56.2–67.4%). Patients with HPV+ OPSCC had significantly greater 2-year OS (83.9% vs. 54.9%; *p* < 0.001) and 2-year DFS (73.5% vs. 45.6%; *p* < 0.001) (Fig. 2A, B). The 2-year OS for patients diagnosed between 2012 and 2016 was not significantly different than the 2-year OS of those diagnosed between 2017 and 2021 (66.4% vs. 75.7%; *p* = 0.105) and neither was the 2-year DFS (60.4% vs. 63.2%; *p* = 0.603) (Fig. 3A, B). When stratified by HPV status, the 2-year OS for HPV+ OPSCC was not significantly different over time (85.8% 2012–2016 vs. 83.0% 2017–2021; *p* = 0.547) and neither was the 2-year DFS (77.2% 2012–2016 vs. 71.7% 2017–2021; *p* = 0.369). A non-significant trend towards improved 2-year OS was observed in the HPV− OPSCC cohort over time (46.5% 2012–2016 vs. 62.7% 2017–2021; *p* = 0.080) with no change in 2-year DFS over time (42.8% 2012–2016 vs. 48.1% 2017–2021; *p* = 0.417) (Fig. 4A, B).

## Discussion

The present study has demonstrated a steady increase in the number of patients with OPSCC within the largest head and neck tertiary referral centre in Ireland over the study period. This is in keeping with observed trends in OPSCC nationally within Ireland which increased from accounting for 13.6% of all head and neck cancers in 1994 to 22.2% of all head and neck cancers in 2014 [11]. Notably, our increase in OPSCC cases locally was driven by an increasing number of HPV+ OPSCC cases. The number of HPV+ OPSCC presentations almost doubled over the study period from 58 HPV+ OPSCC cases between 2012 and 2016 (50.4%) to 117 HPV+ OPSCC cases between 2017 and 2021 (65.4%) (*p* = 0.011). This finding is not unexpected given international trends in the epidemiology of OPSCC. The incidence of OPSCC has been increasing steadily globally, particularly in higher-income countries, largely due to HPV+ OPSCC. It is anticipated this increase will last until at least 2045 [4, 6, 17, 18]. However, the present study’s findings are noteworthy because to our knowledge this trend regarding HPV+ disease is yet to be described in an Irish dataset. While not providing conclusive evidence of causality, the present study supports that an increasing national incidence of OPSCC within Ireland may potentially be driven by an increasing incidence of HPV+ OPSCC.

The majority of patients in this study had HPV-mediated OPSCC (59.5%). Over 200 HPV viral subtypes exist and it is estimated that 65–100% of sexually active adults have been exposed to some form of the virus. Lower risk subtypes (HPVs 6, 11, 13, 32) cause benign pathology such as warts and laryngeal papillomatosis [19]. However, a definitive causal link exists between ‘high-risk’ HPV infection (HPVs 16, 18 31, 33 among others) and OPSCC or anogenital tumours, in particular cervical cancer [19]. Vaccination against ‘high-risk’ carcinogenic strains of HPV is available in order to prevent these cancers. Public and clinician awareness of the link between OPSCC and HPV infection is not as strong as awareness of the link between HPV infection and cervical cancer [20]. This is evidenced by the history of the Irish HPV vaccination program, established in 2010. This program offers students in first year of second level education vaccination against HPV. Notably for the first 9 years of its existence, it is only catered to females. To the surprise of many, worldwide, OPSCC in men alone is more common than cervical cancer [21]. We hope our findings will enlighten the public and clinicians alike to the link between HPV and OPSCC and stress the importance of vaccination among both young males and females.

The proportion of HPV + OPSCC (59.5%) within our study is higher than global estimates (30.8%) [22]. The proportion of HPV-associated disease is known to vary by region internationally with an increased proportion reported in higher-income countries [22]. For example, studies from the United States and Germany have reported their proportions of OPSCC due to HPV as greater than 70% [5, 23]. Most authors describe HPV+ OPSCC patients as typically being male, less likely to smoke or consume alcohol and to be younger than HPV− OPSCC patients with an improved expected OS [7]. This held true in our cohort. However, over half of patients with HPV+ OPSCC had a smoking history in our cohort which is consistent with recent international data [24, 25]. This is important as smoking greatly reduces overall and disease-specific survival in HPV+ OPSCC [8, 26]. Furthermore, the patient population with HPV+ OPSCC is increasing in age internationally and this was again noted over time in our cohort with an increase in mean age from 56.6 to 60.2 years over the study period [27]. In addition, approximately one in five HPV+ OPSCC patients in our cohort were classified as frail using the 5mFI and a similar proportion had a ASA score of 3 or more. Thus, it is evident that our patient population is now more challenging than the classically described middle-aged, non-smoker, non-drinker HPV+ OPSCC patients. This will be an important consideration in developing future multidisciplinary care pathways for these patients. Further evaluations of these trends will be required at a national level.

The overall 2-year OS within our cohort was 72.0% (95% CI 66.4–76.8%). When examined by HPV status, the 2-year OS for HPV+ OPSCC was 83.9% (95% CI 77.4–88.7%) and for HPV− OPSCC 54.9% (95% CI 45.5–63.5%). Despite an increased proportion of HPV+ OPSCC over time, the 2-year OS for all OPSCC patients did not increase in the 2017–2021 group versus the 2012–2016 group. When stratified by HPV status, 2-year OS did not change for either HPV+ or HPV− tumours over time. Our survival outcomes appear similar to an observational OPSCC subgroup analysis from the ARCAGE study although are slightly reduced compared to an analysis of the National Cancer Database (NCDB) in the United States [28, 29]. However, it is notable that when compared to the NCDB analysis, patients in our cohort presented with more advanced T staging, an older age and more significant comorbidities [29]. Additionally, patients treated with palliative intent were excluded from the NCDB analysis and were included within our study. Our findings serve to highlight real-world outcomes managing OPSCC in a unit where patients present with advanced local disease and the presence of a notable proportion of frail and comorbid patients.

As with any study, the present one suffers from limitations. Firstly, owing to the retrospective design, it suffers from inherent biases including selection, confounding and ascertainment bias. Secondly, this is a single unit evaluation of epidemiological trends which may not be generalizable to national data. Nevertheless, it highlights a need to evaluate the population of OPSCC further on a national level given the marked increase in OPSCC seen within Ireland [11]. Finally, in keeping with established practice, our institutional protocol uses P16 as a surrogate marker of HPV infection in OPSCC [15, 16]. Recent evidence has highlighted that almost 10% of cases will have discordant P16 and HPV testing with vastly different expected outcomes [30]. As such, it is possible that a number of cases considered HPV+ or HPV− based on P16 testing may have been misclassified.

In conclusion, our study of 294 patients over 10 years has demonstrated that our OPSCC patient population is changing. There has been a marked increase in case numbers seen locally with shift towards HPV+ OPSCC patients. Considering our findings in light of international trends in the epidemiology OPSCC, it is possible that the increasing national burden of OPSCC within Ireland may be driven by an increase in HPV+ OPSCC. Our study provides local evidence from the largest head and neck cancer centre in Ireland to support this theory. It is important to increase public and clinician awareness of the link between HPV and OPSCC. This data should be used to promote our national vaccination program to both males and females, in order to reduce our national burden of OPSCC.

## Data Availability

Data or code is available on reasonable request from the authors.
